# Antioxidant and Cytotoxic Activity of Hydroethanolic Extract from *Jacaranda decurrens* Leaves

**DOI:** 10.1371/journal.pone.0112748

**Published:** 2014-11-17

**Authors:** Junior Cesar Casagrande, Luis Fernando Benitez Macorini, Katia Avila Antunes, Uilson Pereira dos Santos, Jaqueline Ferreira Campos, Nelson Miguel Dias-Júnior, Andréia Sangalli, Claudia Andrea Lima Cardoso, Maria do Carmo Vieira, Luiza Antas Rabelo, Edgar Julian Paredes-Gamero, Edson Lucas dos Santos, Kely de Picoli Souza

**Affiliations:** 1 Federal University of Grande Dourados, Dourados, Mato Grosso do Sul, Brazil; 2 State University of Mato Grosso do Sul, Dourados, Mato Grosso do Sul, Brazil; 3 Federal University of Alagoas, Maceió, Alagoas, Brazil; 4 Federal University of São Paulo, São Paulo, São Paulo, Brazil; University of Lancaster, United Kingdom

## Abstract

**Background and Purpose:**

Leaves of *Jacaranda decurrens* are used in traditional Brazilian medicine to treat metabolic diseases related to increased reactive oxygen species. The present study evaluated the antioxidant and cytotoxic potential of hydroethanolic extract from the leaves of *Jacaranda decurrens* subsp. *symmetrifoliolata*.

**Experimental Approach:**

Phenolic compounds, flavonoids and saponins were evaluated in an ethanol∶water (80∶20, v/v) extract from the leaves of *Jacaranda decurrens* subsp. *symmetrifoliolata* (E-Jds). The antioxidant activity of E-Jds was investigated by assessing the following: 2,2-diphenyl-1-picrylhydrazyl (DPPH) free radical scavenging activity; protection against 2,2′-azobis (2-amidinopropane) dihydrochloride (AAPH)-induced hemolysis of erythrocytes; *in vitro* and *in vivo* malondialdehyde dosage; and the ability to activate antioxidant enzymes. K562 leukemia cells were used for the cytotoxic evaluation of E-Jds and for the assessment of the cell death profile through flow cytometry.

**Key Results:**

Phenolic and flavonoid compounds were quantified as 14.38% and 2.15%, respectively, of E-Jds. These phenolic and flavonoid compounds proved to be able to scavenge DPPH free radicals with an IC_50_ of 9.3±3.3 µg/mL, to protect up to 50% of erythrocytes against AAPH-induced hemolysis and to reduce *in vitro* and *in vivo* malondialdehyde levels up to 84% and 22%, respectively. E-Jds also increased glutathione peroxidase enzyme activity, with a concomitant decrease in superoxide dismutase and catalase activity, and exhibited dose-dependent cytotoxic activity on K562 erythroleukemia cells with cell death occurring via both late apoptosis and necrosis.

**Conclusions:**

E-Jds exhibits *in vitro* and *in vivo* antioxidant potential, which may be the mechanism mediating the metabolic activities reported in folk medicine. Furthermore, the cytotoxic activity identified in this study contributes with the knowledge of antiproliferative activities that have been described in the literature for the genus *Jacaranda*.

## Introduction

*Jacaranda decurrens* subsp. *symmetrifoliolata* Farias & Proença (Bignoniaceae) is popularly known in Brazil as *carobinha*, *carobinha-do-campo* or *caroba* and is found in Mato Grosso do Sul, Brazil. Different parts of *Jacaranda* spp. are used in folk medicine as teas or potions; the leaves are indicated for diabetes, hyperlipidemia and rheumatic problems [1]. According to Taniyama and Griendling [2], oxidative stress is responsible for the onset and progression of some of these diseases.

Oxidative stress is characterized by an imbalance between the production of reactive oxygen and nitrogen species (ROS/RNS) and the antioxidant defense systems [3]. Antioxidants directly or indirectly delay or inhibit the oxidation process [4], neutralizing the action of free radicals and non-radical species or activating enzyme systems with that capability [5]. If not neutralized, the reactive species cause DNA damage or oxidize lipids and proteins, which then induce cell damage [6] and cause alterations found in various metabolic diseases and disorders [7]. The cellular and molecular mechanisms that underlie the antioxidant capacity of several medicinal plants include the stabilization of the membrane potential, the sequestration of ROS/RNS and the inhibition of lipid peroxidation [8]. These activities have been attributed to biologically active plant components, such as vitamins and phenolic compounds [9].

Another very important pharmacological property related to the secondary metabolites of the medicinal plant is cytotoxic activity, particularly with regard to direct cell death pathways [10], [11]. Currently, several drugs used for cancer treatment are derived from plants, including taxanes, such as paclitaxel, and alkaloids, such as vincristine [12]. The antitumor activity of cytotoxic compounds has been described for plants of the genus *Jacaranda* [13], [14], such as *Jacaranda caroba*, which is popularly used for the treatment of several types of cancer [1].

In this context, the aim of this study was to evaluate the *in vitro* and *in vivo* antioxidant and cytotoxic potentials of the ethanolic extract from the leaves of *Jacaranda decurrens* subsp. *symmetrifoliolata*.

## Materials and Methods

### Plant material and extract preparation

Leaves of *Jacaranda decurrens* subsp. *symmetrifoliolata* (Bignoniaceae), specie not endangered, http://floradobrasil.jbrj.gov.br/2012/index?mode=dp&tid=15684were collected in the Cerrado (Brazilian Savannah), Mato Grosso do Sul state, Brazil, at coordinates 22° 02′ 49.1" S 055° 08′11.4" W, and a voucher specimen was deposited in the DDMS/UFGD herbarium (no. 2322). Authorization of collection of botanical material was granted by Ministério do Meio Ambiente (MMA/ICMBio/SISBIO, number 39451-1).The plant material was dried (45-50°C), crushed and macerated in ethanol∶water (80∶20, v/v) at room temperature for seven days. After this period, the extract was filtered, and the sample underwent two more extractions using the same procedure. After 21 days, the filtrate was concentrated in a rotary vacuum evaporator (FISATOM) and lyophilized, with a 12% yield. The lyophilized hydroethanolic extract, E-Jds, was stored at 4°C and protected from light.

### Dosage of phenolic compounds and total flavonoids

The concentration of phenolic compounds was determined using the method of Meda et al., [15] with some modifications. A 0.5-mL aliquot (200 µg/mL of E-Jds) was mixed with 2.5 mL of Folin-Ciocalteau colorimetric reagent (Dinamica) in a 1∶10 dilution. Subsequently, the aliquot was incubated for 5 min, and then 2 mL of sodium carbonate (14%) was added to the solution. After 2 hours at room temperature, the sample was analyzed in a spectrophotometer at 760 nm. The quantification was performed using a calibration curve with gallic acid as the standard solution (0.4–11.7 µg/mL). The data were subjected to linear regression to obtain the linear equation (y = a+b.x) and to assess the gallic acid (Vetec) concentration determined by absorbance in each sample. Ethanol (80%) was used as a blank, and the results were expressed as mg of gallic acid equivalent per 100 mg of extract. The assay was performed in triplicate.

The concentration of total flavonoids was determined according to the method described by Liberio et al., [16], with modifications, using a solution of 2% aluminum chloride hexahydrate (Vetec) diluted in methanol as a reagent. Two milliliters of E-Jds solution was prepared in methanol∶water (1∶1 v/v) at a concentration of 10 mg/mL. From this solution, 0.5 mL aliquot (200 µg/mL of E-Jds) was added to 4.5 mL of methanol containing 2% aluminum chloride hexahydrate (AlCl_3_.6H_2_O). After 30-min incubation at room temperature, protected from light, the absorbance of the samples was read in a spectrophotometer at 415 nm. To calculate the concentration of flavonoids, a standard curve with quercetin (Sigma-Aldrich) was prepared (0.4–11.7 µg/mL); methanol was used as a blank. The results are expressed as mg of quercetin equivalents per 100 g of extract. The assay was performed in triplicate.

### Saponin determination

A total of 10 mg of E-Jds was dissolved in 2 mL of ethanol, and then 5 mL of boiling water was added to the solution. The solution was stirred vigorously and allowed to stand for 20 min. The qualitative assay was performed in triplicate, and the formation and continued observation of foam in the sample was considered indicative of the presence of saponins [17].

### 2,2-diphenyl-1-picrylhydrazyl (DPPH) free radical scavenging activity

The antioxidant activity of E-Jds was initially evaluated by assessing 2,2-diphenyl-1-picryl-hydrazyl (DPPH; Sigma-Aldrich) free radical scavenging activity, according to Gupta and Gupta, [18]. Control solutions of DPPH at a concentration of 0.11 mM, positive controls [ascorbic acid and butylhydroxytoluene (BHT)], and E-Jds at a concentration of 20 mg/mL diluted in 80% ethanol, were prepared. These solutions were used at various dilutions (1000, 500, 100, 50, 25, 10, 5, 1 and 0.1 µg/mL). The samples were incubated for 30 min, protected from light. Subsequently, absorbance readings were performed in a spectrophotometer at 517 nm. Three independent experiments were performed in triplicate, and the results are expressed as a percentage, using the following equation:

DPPH scavenging (%)  =  [1 – (Absorbance_sample/_Absorbance_control_)] ×100

### Hemolytic activity and protection against 2,2′-Azobis(2-amidinopropane) dihydrochloride (AAPH)-induced hemolysis

After approval by the Research Ethics Committee (*Comitê de Ética em pesquisa* – CEP) of the Grande Dourados University Center, Brazil (Process number CEP 123/12), 5 mL of peripheral blood was collected from healthy donors, stored in tubes containing sodium citrate and subsequently centrifuged at 2000 rpm (700 *g*) for 5 min. After centrifugation, the buffy coat was removed from the plasma. The remaining erythrocytes underwent three washes with saline (0.9% NaCl) at 1500 rpm (350 *g*) to remove possible interferents, with the supernatant discarded after each wash cycle. Subsequently, a solution of 10% erythrocytes was prepared in saline (He).

The protection against lipid peroxidation was assessed by the hemolysis protection method induced by 2,2′-azobis(2-amidinopropane) dihydrochloride (AAPH; Sigma-Aldrich) described by Valente et al., [19]. The erythrocytes were pre-incubated at 37°C for 30 min in the presence of different concentrations of ascorbic acid or E-Jds (50, 75, 100 and 125 µg/mL). Then, a solution of 50 mM AAPH was added. Total hemolysis was induced by incubating He with distilled water. Basal hemolysis caused by E-Jds was assessed by incubating He with the extract without the presence of a hemolysis inducer, and the negative control was assessed in He incubated only with 0.9% NaCl. After adding AAPH, aliquots were collected at 60, 120, 180 and 240 min and read at 540 nm. The results were expressed by multiplying the absorbance values of samples treated with E-Jds and ascorbic acid by 100 and dividing the result by the total hemolysis absorbance. Three independent experiments were performed in triplicate.

### Dosage of malondialdehyde (MDA)

***In vitro***. A suspension of 20% He was used to assess the protective effects of E-Jds against lipid peroxidation. The erythrocytes were pre-incubated at 37°C for 30 min, with different concentrations of E-Jds or ascorbic acid (50, 75, 100 and 125 µg/mL). A sample of 1% ethanol was used as the negative control. Then, 50 mM AAPH was added to the erythrocyte solutions, which were then incubated at 37°C for 3 h, with periodic agitation. Subsequently, the samples were centrifuged at 2000 rpm for 5 min, and an aliquot of 0.5 mL of supernatant was added to 0.5 mL of 20% trichloroacetic acid (Vetec). From this mixture, a 0.5-mL aliquot was removed and added to tubes containing 1 mL of 10 nM thiobarbituric acid/TBA reagent (Merck) and incubated at 96°C for 45 min. Samples were then kept at room temperature for 15 min, followed by the addition of 3 mL of butanol, with subsequent agitation and centrifugation at 3000 rpm, as described by Campos et al., [20]. The supernatant absorbance was read in a spectrophotometer at 532 nm, and three independent assays were performed in triplicate. For this assay, 500 µL of 20 mM MDA solution was used as a standard. MDA levels of samples were expressed in nmol/mL, obtained according to the following equation:

MDA  =  Absorbance_sample_ × (20×220.32/Absorbance_standard_)

***In vivo***. The Ethics Committee of the Federal University of Grande Dourados approved all procedures, under protocol no. 022/2012. Wistar rats (n = 12) were fed a 66% fructose-rich diet for a period of three months to increase blood glucose levels, which can induce oxidative stress [21]. Subsequently, the rats were treated for another two months with water administered orally by gavage (n = 6, Control Group) or with E-Jds at a concentration of 400 mg/Kg (n = 6, Group E-Jds), in addition to the above diet. After this period, the animals were sacrificed by decapitation under anaesthetic, and the blood from the vascular trunk of the neck was collected and centrifuged at 3000 rpm for 5 min. Serum was used to measure serum malondialdehyde levels. For this purpose, 200 µL of serum was incubated for 45 min at 96°C with 1 mL of 10 nM thiobarbituric acid (TBA). The samples were kept at room temperature for 15 min, followed by the addition of 3 mL of butanol, with subsequent agitation and centrifugation at 3000 rpm for 5 min. The supernatant absorbance was read on a spectrophotometer at 532 nm, as described in section 2.6.1.

### Enzyme activity assessment

To assess the effects of E-Jds on REDOX balance, the activity of enzymes, such as glutathione peroxidase (GPx), superoxide dismutase (SOD) and catalase (CAT), were measured in erythrocyte lysates (25, 50, 75, 100, 125, 150, 175 and 200 µg/mL of E-Jds in samples previously diluted in 50 mM PBS). In all assays, the erythrocyte dilution was used at a 1∶20 ratio (in 25 mmol/L of potassium phosphate, pH 7.5, supplemented with 0.1% bovine serum albumin and 1 mmol/L EDTA). All enzyme assays were performed in microplates (Multiskan GO Microplate Spectrophotometer; ThermoScientifc, Vantaa, Finland). Two independent experiments were performed in duplicate.

#### Glutathione peroxidase activity

E-Jds (10 µL), at final concentrations of 25, 50, 75, 100, 125, 150, 175 and 100 µg/mL, and 20 µL of the previously diluted erythrocyte lysate were plated. Subsequently, the reaction mixture was incubated for 20 min at 37°C. GPx activity was determined using a colorimetric method, as described by Paglia and Valentine, [22], with modifications.

#### Superoxide dismutase activity

Ten microliters of E-Jds at the final concentrations previously described and 20 µL of previously diluted erythrocyte lysate were sequentially added to a 96-well microplate. The reaction mixture was incubated for 20 min at 37°C. SOD activity was determined using the Fluka commercial kit (Sigma-Aldrich, Seelze, Germany), according to the manufacturer's instructions. The data are expressed as total international unit (IU) normalized to hemoglobin concentration in mg/mL. Hemoglobin levels were evaluated using a commercial kit (Labtest, Belo Horizonte, Brazil).

#### Catalase activity

We sequentially plated 10 µL of E-Jds at the final concentrations previously described and 20 µL of previously diluted erythrocyte lysate. After incubation for 20 min at 37°C, CAT activity was assessed spectrophotometrically by the rate of H_2_O_2_ decomposition, according to the method described by Xu et al., [23], with modifications for microplates. The enzyme activity is expressed as µmol/min/mL and normalized to hemoglobin concentrations in mmol/L. The data were normalized to hemoglobin levels, and the activity is expressed as µmoL/min/mL/mMHb.

### Cell line and culture conditions

The K-562 erythroleukemia cell line was ordered from the American Type Culture Collection (ATCC CCL-243). The K-562 cell line was grown in suspension in RPMI 1640 medium (Cultilab, Brazil), supplemented with 10% fetal bovine serum (FBS; Cultilab, Brazil), 100 U/mL of penicillin and 100 µg/mL of streptomycin in a humidified atmosphere at 37°C in 5% CO_2_.

#### Cytotoxicity assay and cell death profile

Cytotoxic activity and the cell death profile were evaluated according to the method described by Paredes-Gamero et al., [24], with minor modifications. Cells were plated on 96-well plates (10^5^ cells/mL) and grown in medium containing 10% FBS in the absence or presence of E-Jds (1–1000 µg/mL) for 24 h. Then, K562 cells were washed with PBS and resuspended in a buffer for annexin labeling (0.01 M HEPES (pH 7.4), 0.14 M NaCl and 2.5 mM CaCl_2_). Cell suspensions were labeled with annexin-FITC and propidium iodide (PI; Becton Dickinson, USA), according to the manufacturer's instructions. Cells were incubated at room temperature for 15 min and analyzed by flow cytometry on a FACSCalibur cytometer (Becton Dickinson, USA) using the CellQuest software (Becton Dickinson, USA). A total of 10,000 events were collected per sample.

#### Caspase-3 activity

Caspase-3 activity was assessed by flow cytometry, according to the method described by Moraes et al., [25], with minor modifications. K562 cells were plated on 24-well plates (10^5^ cells/mL), grown in medium containing 10% FBS and treated with E-Jds (200 and 1000 µg/mL) for 24 h. After treatment, cells were centrifuged, washed and fixed with 2% paraformaldehyde in PBS for 30 min. Then, the cells were centrifuged and washed with glycine (0.1 M) in PBS, permeabilized with 0.01% saponin for 30 min and blocked with PBS containing 1% BSA for 30 min at room temperature. Subsequently, cells were incubated with a monoclonal FITC-conjugated anti-caspase-3 antibody (BD-Pharmingen, San Diego, CA, USA) in the dark at room temperature for 40 min. Fluorescence was analyzed in a FACSCalibur cytometer (Becton Dickinson, USA) using the CellQuest software (Becton Dickinson, USA); 10,000 events were collected per sample.

#### Mitochondrial membrane potential (ΔΨ) assessment

Changes in ΔΨ were assessed using JC-1 (5,5′,6,6′-tetrachloro-1,1′,3,3′-tetraethylbenzimidazolylcarbocyanine iodide; Molecular Probes, Eugene, OR, USA) dye incorporation, according to the method described by Moraes et al., [25]. JC-1 is a cationic dye that shows membrane potential-dependent accumulation in mitochondria, which is indicated by a shift in fluorescence emission from green (∼520 nm) to red (∼590 nm). Thus, cells stained red indicate a higher ΔΨ, while cells stained green indicate a reduced ΔΨ. For this purpose, K562 cells were seeded on 24-well plates (10^5^ cells/mL), grown in medium containing 10% FBS and treated with E-Jds (200 and 1000 µg/mL) for 24 h. Then, the cells were centrifuged and incubated with JC-1 (1 mg/mL) for 15 min at room temperature. Fluorescence was analyzed in a FACSCalibur cytometer (Becton Dickinson, USA) using the CellQuest software (Becton Dickinson, USA; 10,000 events were collected per sample.

### Statistical analyses

The data are shown as the mean ± standard error of the mean and were subjected to one-way analysis of variance (ANOVA) followed by Dunnett's test for comparison of more than two groups or a t-test for comparison between two groups. The results were considered significant when P<0.05.

## Results and Discussion

Exogenous antioxidants are important for maintaining health; among them are the phenolic compounds [26] and flavonoids [27], which were quantified in E-Jds as being 14.38±5 mg of gallic acid/100 mg of extract and 2.15±3 mg of quercetin/100 mg of extract, respectively, thus representing a finding similar to that observed by Carvalho et al., [28] for the subspecies *Jacaranda decurrens* Cham. Additionally, saponins were not found in E-Jds.

Considering the presence of potentially antioxidant substances, an *in vitro* evaluation of the DPPH free radical scavenging activity of E-Jds was performed at different concentrations. The 50% inhibitory concentration (IC_50_) and the maximal DPPH free radical scavenging activity of E-Jds and the controls used are shown in Table 1. The IC_50_ of E-Jds was approximately half that of BHT and five times higher than that of ascorbic acid.

**Table 1:** IC_50_ and maximum activity of DPPH free radical scavenging of standard antioxidants and the hydroethanolic extract from the leaves of *Jacaranda decurrens* subsp. *symmetrifoliolata* (E-Jds).

Sample	IC_50_ (µg/mL)	(n)	Maximum inhibition	
			%	µg/mL
Ascorbic acid	1.8±0.4	3	92±0.8	10
BHT	18.3±4.5	3	94±1.3	500
E-Jds	9.3±3.3	3	92±0.7	50

BHT  =  butylhydroxytoluene

The presence of phenolic compounds in E-Jds can be directly related to the scavenging of DPPH radicals. The structures of phenolic compounds contain hydroxyl groups, which can capture the reactive species [26]. Among these compounds are the flavonoids, which, when ingested, are transformed to phenolic acids capable of neutralizing free radicals. According to Pietta [29], the antioxidant protection provided by these compounds results from increased plasma antioxidant capacity and the preservation of vitamin E and polyunsaturated fatty acids in the erythrocyte membrane.

Several flavonoids, such as luteolin [30] and jacaranone [31], triterpenes, ursolic acid and oleanolic acid [32], have been identified in the genus *Jacaranda*, and their antioxidant activities have been described [33], [34], [35], [36].

**Figure 1:**
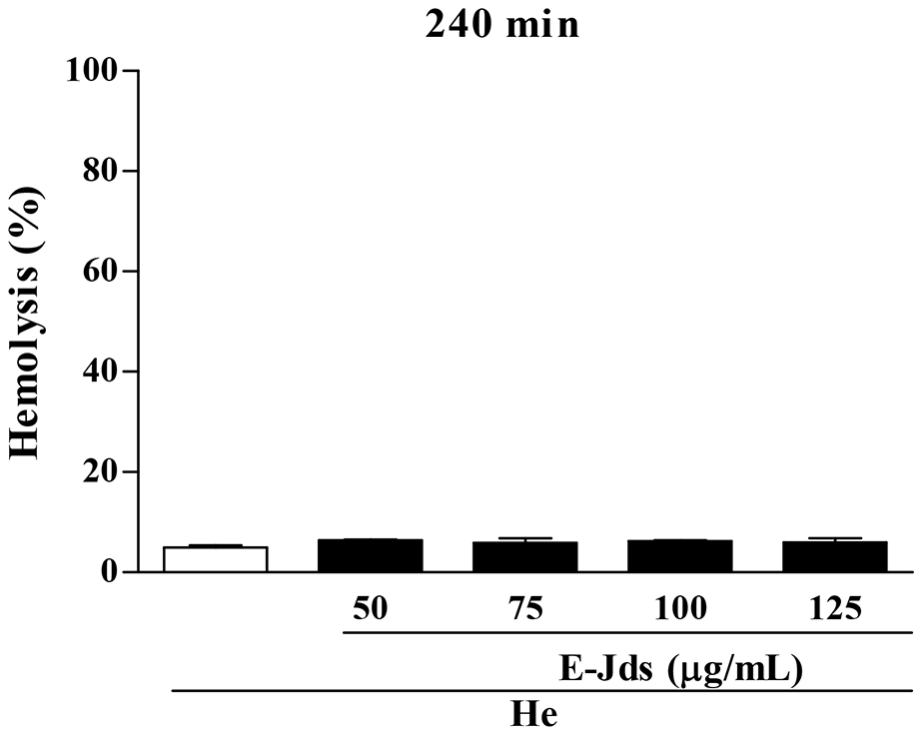
Hemolysis assessment at 2.5% (He) incubated with different concentrations of E-Jds for 240 min. E-Jds  =  hydroethanolic extract from the leaves of *Jacaranda decurrens* subsp. *Symmetrifoliolata*. The data no are statistically different.

After the initial confirmation of antioxidant activity, the effect of E-Jds in human erythrocytes was evaluated. E-Jds did not induce hemolysis after incubation for 240 min (Figure 1), indicating that, at the concentrations evaluated, the extract is not toxic to normal cells.

**Figure 2:**
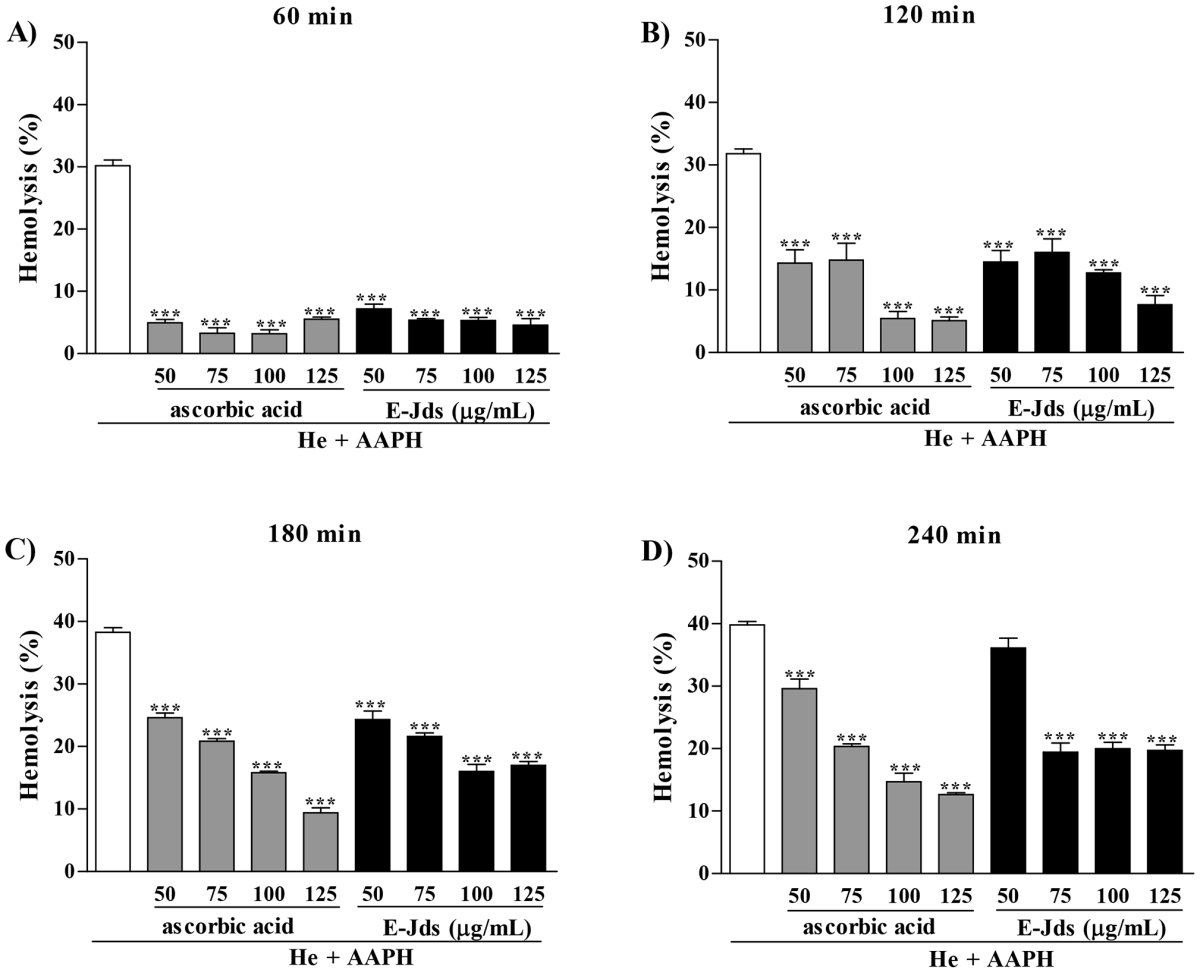
Hemolysis assessment at (A) 60, (B) 120, (C) 180 and (D) 240 min after addition of AAPH in erythrocytes at 2.5% incubated with different concentrations of ascorbic acid and E-Jds. *** P<0.001 *vs.* HE+AAPH.

Next, the ability to protect against AAPH-induced lipid peroxidation of human erythrocytes was evaluated. During the 240-min incubation with the hemolysis inducer AAPH, E-Jds showed activity similar to ascorbic acid, protecting cells from cell lysis, except for the latest time point and the lowest concentration evaluated (Figure 2). The lipid peroxidation decrease promoted by E-Jds, as indicated by hemolytic reduction, was 50% at the highest dose and the latest time point.

**Figure 3:**
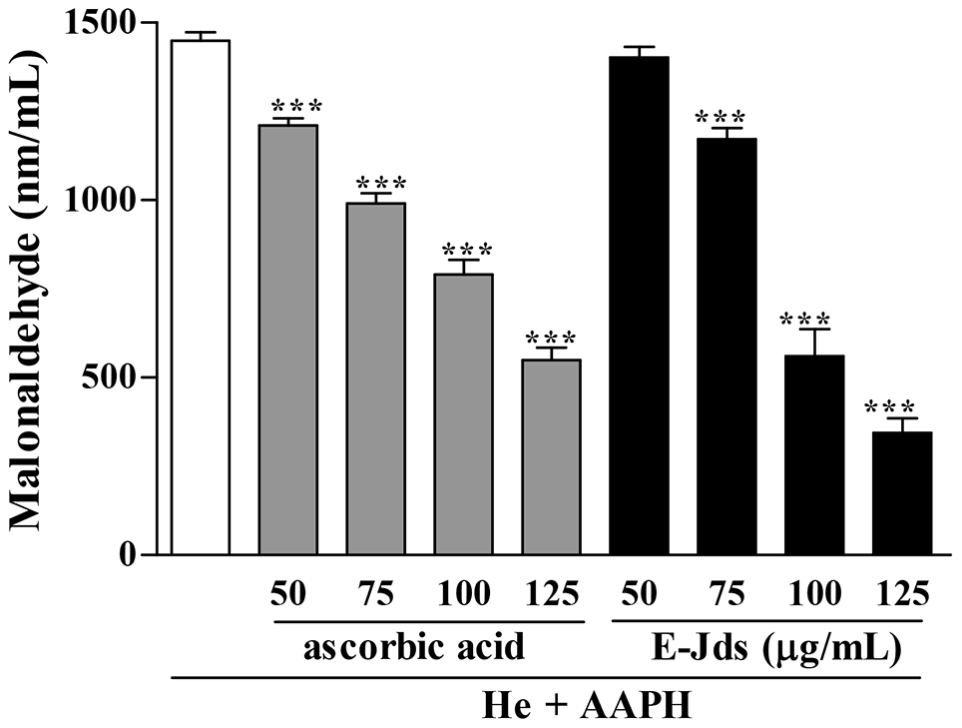
AAPH-induced lipid peroxidation of erythrocytes. Concentration of malondialdehyde (MDA) at 180 min after adding the AAPH hemolysis inducer in 2.5% erythrocytes incubated with different concentrations of ascorbic acid and hydroethanolic extract of *Jacaranda decurrens* subsp. *symmetrifoliolata* (E-Jds) leaves compared with 5% He + AAPH. *** P < 0.001 *vs.* He+AAPH.

The peroxidation of polyunsaturated fatty acids at the cell membrane, induced by an excess of free radicals [37], causes damage and cell death [38] with the release of malondialdehyde (MDA), a byproduct derived from lipid peroxidation [6]. After a 180-min incubation of erythrocytes with E-Jds, a dose-dependent reduction of MDA generation was observed (Figure 3). A reduction of up to 84% of MDA was observed in samples of erythrocytes incubated with the highest concentration of the extract. E-Jds provided a protective effect against lipid peroxidation similar to that of ascorbic acid, which is a vitamin with antioxidant capacity as demonstrated by decreased lipid peroxidation and MDA production [39].

Antioxidants that protect against cell damage may support the treatment of various diseases, given that the excess of non-neutralized reactive species is related to the etiology and progression of hypertension, hyperlipidemia, diabetes mellitus [2] and cancer [40], among others.

**Figure 4:**
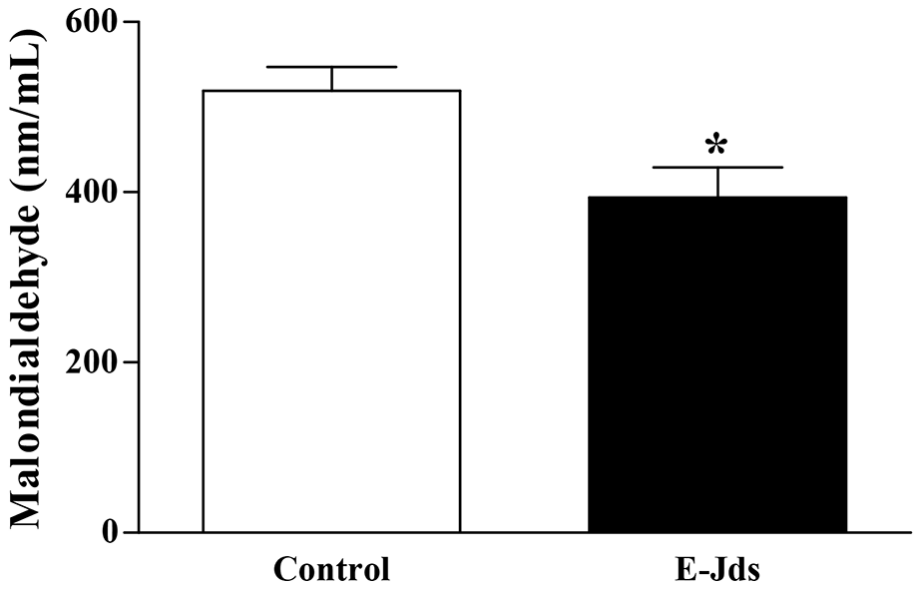
Malondialdehyde serum concentration in Wistar rats fed a high-fructose diet (66% fructose), treated with water (control) or hydroethanolic extract from the leaves of *Jacaranda decurrens* subsp. *symmetrifoliolata* (E-Jds, 200 mg/Kg), for 60 days by gavage. *P<0.05 *vs.* E-Jds.

In this scenario, with positive results for *in vitro* antioxidant activity, we sought to investigate the activity of E-Jds using an *in vivo* model. Wistar rats subjected to a 66% high-fructose diet treated with E-Jds showed a 22% reduction in serum MDA levels compared with animals receiving only water (Figure 4).

The reduced serum MDA shows an increase in its *in vivo* antioxidant capacity, as evidenced by the decrease in lipid peroxidation. Taken together, these data indicate that E-Jds may have a beneficial effect on several diseases [41].

**Figure 5:**
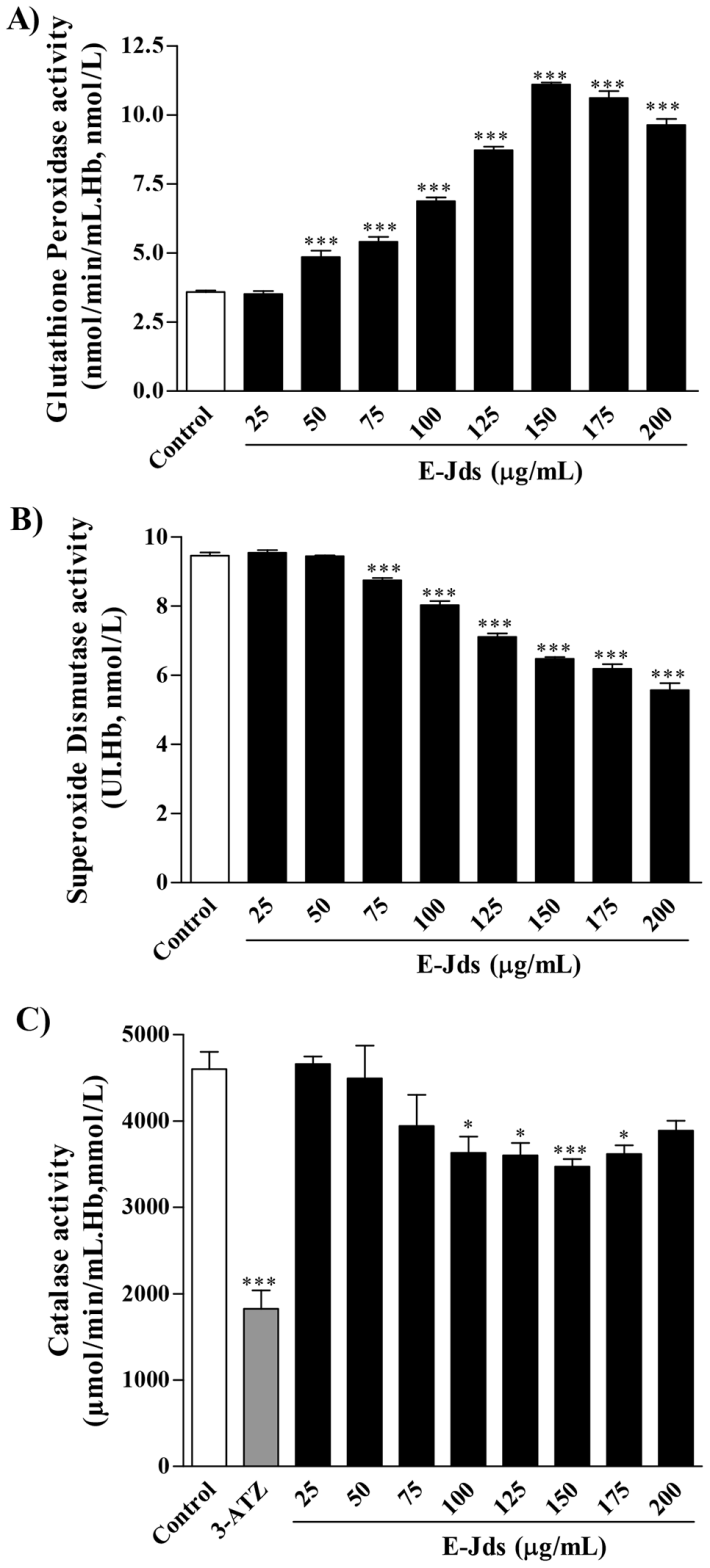
Activity of the antioxidant enzymes glutathione peroxidase (A), superoxide dismutase (B), and catalase (C) in human erythrocyte lysates incubated with E-Jds at different concentrations. * P<0.05 and *** P<0.001 *vs.* Control.

After the *in vitro* cell protection effect and the ability to reduce the generation of MDA *in vivo* exerted by E-Jds were confirmed, the activities of the antioxidant enzymes superoxide dismutase, catalase and glutathione peroxidase were evaluated in erythrocyte lysates. E-Jds increased the enzyme activity of glutathione peroxidase and subsequently reduced the activity levels of superoxide dismutase and catalase (Figure 5).

Protection against erythrocyte cell damage may correlate with increased glutathione peroxidase activity, which has antioxidant effects against organic peroxides through glutathione oxidation [42]. Flavonoids isolated from the genus *Jacaranda* [30], including luteolin, increase the activity of glutathione peroxidase [43] in addition to inhibiting the action of another enzyme, xanthine oxidase, which catalyzes the oxidation of hypoxanthine to xanthine and generates superoxide anion during the process. The reduced production of xanthine, together with the fact that phenolic compounds have the ability to scavenge superoxide, leads to a reduction in the concentration of the free radical superoxide [44], which is dismutated by SOD [45] to form hydrogen peroxide (H_2_O_2_). The reduced formation of H_2_O_2_, together with increased activity of glutathione peroxidase, which metabolizes H_2_O_2_, reduces the concentration of this reactive species required for the activity of catalase. Thus, the reductions in SOD and CAT enzyme activities observed in this study might be due to increased GPx activity.

**Figure 6:**
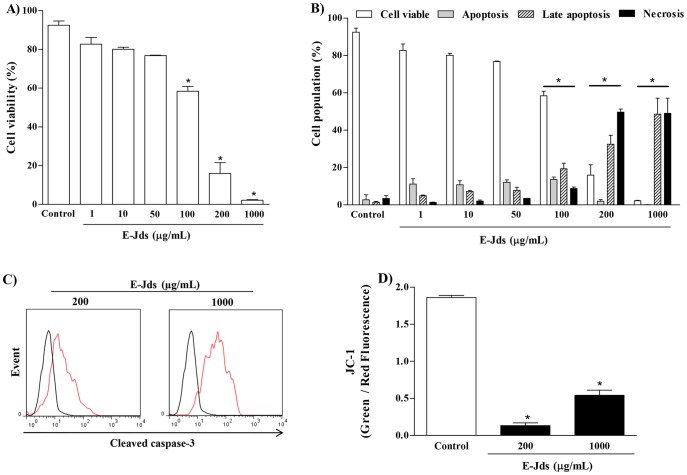
(A) Cell viability of K562 erythroleukemia cells incubated with different concentrations of E-Jds, (B) cell death profile, (C) caspase-3 activity and (D) mitochondrial membrane potential. * P<0.05 *vs* Control.

Considering the concentration-dependent antioxidant and cytotoxic action of jacaranone [46], a compound also isolated from the leaves of *Jacaranda mimosifolia* [31], the cytotoxic activity of E-Jds was studied in K562 erythroleukemia cells. There was a concentration-dependent cytotoxic activity against the tumor cells studied (Figure 6A), and the reduction in viability occurred through late apoptosis and necrosis (Figure 6B), as shown by the activation of caspase-3 (Figure 6C) and the lowered mitochondrial membrane potential (Figure 6D).

Several species of *Jacaranda* have been described as having antiproliferative activity. Ogura et al. [47] described the biological activities of *Jacaranda caucana* related to cytotoxic and anticancer activity in lymphocytic leukemia. Recently, Yamasaki et al. [13] found that oil from *Jacaranda mimosifolia* seeds promotes apoptosis in human HL-60 leukemia cells. The antioxidant and cytotoxic activities observed in this study may be due to the presence of phenolic compounds. At high concentrations, the phenolic compounds can become pro-oxidants and change the redox balance of tumor cells [46]. An increase in intracellular ROS can promote loss of mitochondrial membrane potential and apoptosis via activation of caspase-3 [11]. The present study showed a reduction in the mitochondrial membrane potential and an increase in caspase-3 activation, with a decrease in cell viability and an increase in cell death through late apoptosis at the highest concentrations of E-Jds evaluated. The cytotoxic action observed in this study corroborates that observed by De-Almeida et al., [48] for the hydroethanolic extract of *Jacaranda puberula* Cham in K562 erythroleukemia cells, although the mechanism of death indicated in that study was apoptosis. E-Jds also induced death through necrosis, an alternative mode of death for cells resistant to apoptosis that occurs with some conventional chemotherapeutic agents [49].

## Conclusions

The results of the present study show that the hydroethanolic extract of *Jacaranda decurrens* subsp. *symmetrifoliolata* leaves has *in vitro* and *in vivo* antioxidant activity and cytotoxic activity against erythroleukemic cells that is induced by both late apoptosis and necrosis. This allows for new perspectives on its use in situations involving oxidative stress and cell proliferation.
